# Restoring comparative expertise to improve translation of animal models

**DOI:** 10.1242/dmm.053172

**Published:** 2026-08-26

**Authors:** K. C. Kent Lloyd

**Affiliations:** Department of Surgery, School of Medicine, Mouse Biology Program, and NAMs Testing Center, University of California Davis, Davis, CA 95618, USA

**Keywords:** Comparative pathology, Animal models, New approach methodologies, Translational research

## Abstract

Animal models remain indispensable in biomedical research, particularly for discovering disease mechanisms within intact living organisms. Yet confidence in their human translational relevance has been weakened by apparent failures of preclinical findings to predict clinical outcomes. Although often attributed to species differences, such failures also reflect correctable problems in model selection, experimental design, endpoint choice and phenotype interpretation. A key, but overlooked, contributor is the erosion of comparative expertise in pathobiology and clinical medicine needed to relate animal phenotypes to human disease across species, strain, and developmental, physiological and pathological contexts. This expertise gap can lead investigators to mismatch models to biological questions, misinterpret background abnormalities as engineered phenotypes or extend mechanism-focused models beyond the questions they are suited to answer. New approach methodologies (NAMs), such as *in vitro* and *in silico* models, provide important complementary tools, but do not eliminate the need for whole-organism models or resolve this interpretive problem. Ensuring the reliability of biomedical research will require preserving well-justified animal models where whole-organism systems biology remains necessary, integrating validated NAMs only where they are fit for purpose, and rebuilding the comparative expertise needed to interpret both model types rigorously.

## Teaser

**Summary:** To ensure the translational success of disease models, we must ensure that phenotypes are interpreted with the comparative pathobiology and clinical expertise needed to connect animal biology to human disease.

## Animal models and the translational relevance debate

Animal models have long been central to biomedical research because they allow investigators to study disease within the context of an intact living organism (Justice and Dhillon, 2016; Brown et al., 2018; Lloyd, 2024). Their central strength lies in revealing biological relationships when investigators do not yet know which cells, organs or pathways matter, supporting both mechanistic discovery and hypothesis testing across biological scales, from genes to whole-organism physiology (Capecchi, 2005; Brown et al., 2018; Lloyd, 2024). Yet confidence in the translational relevance of these findings is increasingly challenged by concerns that animal findings do not always predict human outcomes (van der Worp et al., 2010; Mak et al., 2014; Justice and Dhillon, 2016). This leads researchers to question why their relevance to human disease is sometimes uncertain.

This skepticism persists despite a substantial track record, including a majority of Nobel Prizes in Physiology or Medicine (Baptista et al., 2021; Capecchi, 2005). It is fueled by reported translational failures attributed to discordances between animal and clinical outcomes, attrition above 90% for drug candidates in clinical development, reproducibility concerns and publication bias (Perel et al., 2007; van der Worp et al., 2010; Sena et al., 2010; Leenaars et al., 2019; Ineichen et al., 2024; Hackam and Redelmeier, 2006; Mak et al., 2014; Cook et al., 2014; Hay et al., 2014). These concerns have driven US Food and Drug Administration (FDA), National Institutes of Health (NIH), European Medicines Agency (EMA), and Commonwealth Scientific and Industrial Research Organisation (CSIRO) initiatives to cite species differences as justification to reduce animal use in preclinical research and catalyze adoption of new approach methodologies (NAMs), such as *in vitro* and *in silico* models (FDA, 2025; NIH, 2025; EMA, 2025; CSIRO, 2023). But species differences are not the whole story. Some failure reflects mechanism-focused models being misapplied to drug efficacy questions, and much reflects experimental design, reproducibility, trial design and regulatory factors unrelated to the animal model itself (Paul et al., 2010; Collins, 2011).

What, ultimately, is the origin of these failures in experimental planning, design and interpretation? A pernicious shortage of comparative pathobiology and clinical medicine expertise capable of interpreting animal phenotypes has created a severe gap in translating research findings from animals to the human condition (Cardiff et al., 2008). Investigators unfamiliar with strain-specific pathology can mistake spontaneous background abnormalities for engineered phenotypes. For example, in common strains such as C57BL/6J mice, incidental brain lesions or age-related degenerative changes can be misread as experimentally induced neurological phenotypes (Tarrant et al., 2020; Finesso et al., 2023). Similar findings can be misinterpreted in other animal species as well, including progressive nephropathy in F344 rats (Travlos et al., 2011) and multi-organ inflammatory changes in Goettingen minipigs (Jeppesen and Skydsgaard, 2015). Although one should always use a non-engineered background strain as a negative control, they are not foolproof. Background lesions might or might not be perfectly penetrant (that is, present in every animal), and an engineered mutation might worsen a background lesion (e.g. drug-induced exacerbation of progressive nephropathy in F344 rats). This gap in knowledge and interpretation has emerged gradually over the past two decades and has now become a crisis as retirements, funding contraction and the elimination of training positions eroded the pipeline of experts in comparative pathology and medicine.

## The erosion of comparative expertise

Modern pathobiology has deep comparative roots. Rudolf Virchow emphasized mechanisms shared between human and animal disease (Saunders, 2000), and this tradition shaped modern clinical medical education, including William Osler's comparative approach to pathology, underpinning major discoveries in infectious disease, immunology and physiology (Bliss, 2007). Thanks in large part to Calvin Schwabe (Schwabe, 1984), for much of the 20th century, investigators trained across veterinary pathology, human pathology and laboratory animal biology interpreted experimental phenotypes within this cross-species framework. This training is essential because model validity depends not only on genetic or physiological similarity, but on accurately recognizing disease manifestations in the model organism itself.

**Fig. 1.**
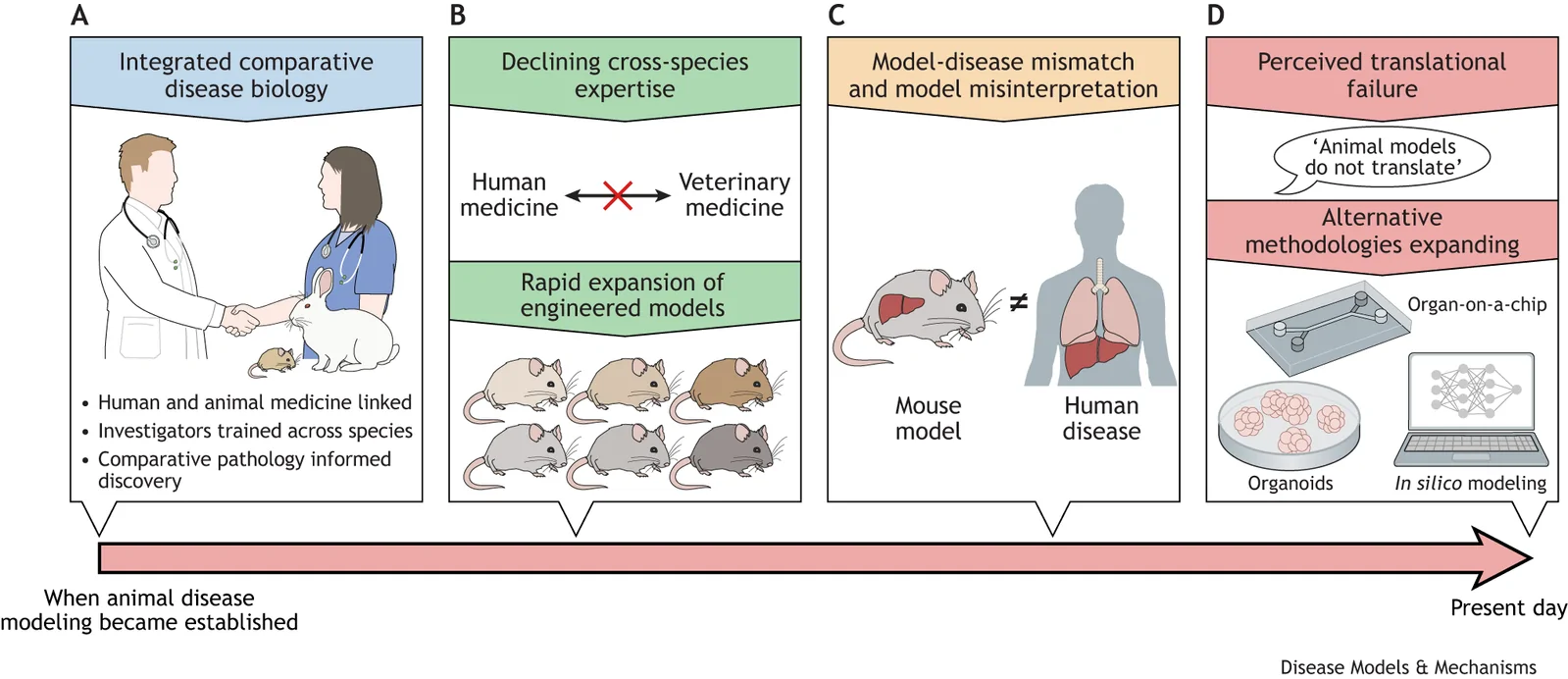
**Historical drivers of the perceived translational gap between animal models and human disease.** (A) Early biomedical research operated within a comparative framework in which human and veterinary medicine were closely linked and investigators were trained to interpret disease processes across species. (B) Over time, increasing institutional separation between medical and veterinary disciplines reduced the number of researchers with cross-species expertise, even as genetically engineered mouse models rapidly expanded in number and complexity. (C) When experimental models are developed or interpreted without sufficient comparative pathobiological context, discrepancies between model phenotypes and human disease might arise. (D) These mismatches can then be perceived as failures of the model organism itself, contributing to the narrative that animal models translate poorly and motivating calls for alternative methodologies. This challenge is also an opportunity to recognize the need to restore comparative expertise and to encourage engagement between the new approach methodology (NAM) and animal model communities.

Over time, the separation of human and veterinary medical training, increasing biomedical specialization, and the rise of molecular and genetic approaches steadily narrowed this comparative framework (Schwabe, 1984; Woods and Bresalier, 2014) (Fig. 1). Concerns about the resulting shortage of comparative expertise surfaced in the early 2000s as genetically engineered mouse models expanded dramatically. Training opportunities contracted or were eliminated, and senior experts approached retirement without a proportional expansion of the next generation (Fox et al., 2006; Schofield et al., 2012; Barthold et al., 2016). Today, the responsibility for interpreting phenotype increasingly falls to research scientists who might have deep expertise in molecular biology and genetics but are unlikely to have had formal pathology or clinical training in recognizing spontaneous, strain-specific, age-related or experimentally induced disease.

This gap has widened just as the expansion of mouse models made expert interpretation more necessary, not less. Gene-targeting technologies and large-scale efforts, such as the International Mouse Phenotyping Consortium, have made it possible to generate and phenotype mutant mice at unprecedented scale (Capecchi, 2005; Dickinson et al., 2016; Brown et al., 2018; Lloyd, 2024). Although a mouse line can validly model a pathway, cell type or restricted disease feature, it will not reproduce the full human syndrome. This can be particularly difficult when attempting to characterize phenotypes associated with incomplete penetrance and variable expressivity due to genetic haploinsufficiency (Cooper et al., 2013). When that distinction is not made explicit, a model built to interrogate mechanism can be criticized as a failed clinical predictor, or a background lesion can be mistaken for an engineered phenotype. These are errors of interpretation, not of biology. The relevant question is, therefore, not whether a model perfectly replicates human disease (none do), but whether it is fit for purpose, its domain of inference is clearly defined, and its phenotype is interpreted within the appropriate comparative biological context (Justice and Dhillon, 2016).

Empirical evidence shows that comparative expertise measurably changes how animal models are interpreted. Large-scale phenotyping found that histopathology revealed previously unrecognized disease-related phenotypes in ∼14% of mouse lines that appeared normal by routine screening and added interpretive value in more than half of lines with known abnormalities (Adissu et al., 2014). Histopathology has, likewise, proven essential for detecting and classifying myopathies in mouse models of skeletal muscle disease that tests of muscle and bone strength alone missed (Vogel et al., 2021). Together, these findings show that accurate model interpretation depends on expertise in comparative pathobiology and a complete mechanistic understanding of disease pathobiology, not merely on technical skill in model generation. The epistemic value of *in vivo* models is tightly linked to the availability of experts able to compare and interpret phenotypes across biological scales.

## The comparative expertise hypothesis

Together, these observations support a testable hypothesis that some translational failures attributed to inherent species differences may instead arise from insufficient comparative expertise in pathobiology and clinical medicine during experimental design and interpretation. Designing a meaningful animal model of disease requires alignment among the molecular mechanisms of the disease being modeled, its pathological and clinical manifestations in humans, and the normal biological and pathological features of the model organism. Misalignment among these domains – such as modeling the wrong disease stage; emphasizing endpoints of limited clinical relevance; overlooking background strain effects; misclassifying adaptive responses as pathology; neglecting age and sex effects; failing to consider dose, route and treatment duration effects; or conflating primary and secondary lesions – can produce an apparent model–disease mismatch that is later attributed to the animal system itself, when the underlying problem may be interpretive rather than ontological.

Species differences remain real and important, but that fact strengthens, rather than weakens, the case for comparative expertise. The more research relies on *in vivo* systems, the more essential it becomes to know which biological processes are conserved, divergent or phenotypically analogous but not identical across species. This holds whether the model is a mouse, a zebrafish or a fly. Comparative expertise is, therefore, not an optional supplement to animal research. It is part of what makes that research scientifically interpretable.

This hypothesis also generates testable predictions. Retrospective analyses could examine whether translational outcomes differ between studies that involve comparative pathology and clinical expertise and those that do not, whether pathology-based phenotyping improves reproducibility, whether institutions with strong cross-species pathology infrastructure produce more interpretable disease models, and whether explicit fit-for-purpose model definitions improve downstream translation. Such studies would help determine whether reinvestment in comparative expertise measurably increases the scientific value of *in vivo* models.

## NAMs as complementary tools, not substitutes or replacements

The comparative expertise hypothesis, albeit indirect and not yet proven, has important implications for ongoing debates surrounding NAMs. Organoids, microphysiological systems, organ-on-a-chip platforms and computational disease models offer powerful new tools for studying human biology. They can provide human-specific material, tighter experimental control, higher throughput, and readouts that are difficult or impossible to obtain in whole-animal studies. Once validated for their intended use against appropriate human, clinical and, where relevant, *in vivo* benchmarks, these tools have major advantages and should be embraced.

However, the strengths of NAMs do not make them wholesale replacements for *in vivo* models. Organoids and organ-on-a-chip platforms can recapitulate selected aspects of tissue architecture and organ interfaces, but they remain engineered simplifications that typically lack the full systemic context of immunity, vascularization, innervation, metabolic, endocrine signaling and whole-body physiology. Computational models, similarly, are limited by the quality, scope and biological completeness of the data used to build them (Kim et al., 2020; Low et al., 2021; Vunjak-Novakovic et al., 2021).

In other words, NAMs are strongest when the relevant pathobiology and clinical effects are already sufficiently understood to be represented, constrained and measured in a reduced system. They are less well suited to revealing unknown multiscale biology that only becomes apparent in the whole organism. Comparative expertise has a role here too. Judging how faithfully a NAM recapitulates the disease features seen in a validated *in vivo* model is, itself, a comparative exercise. Replacing animal models with NAMs alone would, therefore, not solve the underlying interpretive problem. It would simply move that problem into a new experimental domain. The most scientifically productive path is not replacement rhetoric, but principled integration of complementary methods (Lloyd, 2025; Bogani et al., 2026).

## Reintegrating comparative expertise into translational science

Animal models remain essential for biomedical research because NAMs cannot yet replace all the features of an intact *in vivo* system. Despite efforts to link multiple organ-on-a chip platforms (Mitchell et al., 2026), it is likely that they never will because of the complexity of human diseases, as can be appreciated by notable heterogeneity even in diseases driven by a single-gene defect, such as cystic fibrosis or α1-antitrypsin deficiency, let alone a more complex multifactorial disease, such as metabolic syndrome. In this context, comparative expertise will play an increasingly significant role in translational success when using animal models and NAMs. Strengthening this discipline should, therefore, become a practical priority for the biomedical research enterprise. This gap is more acute in academia than in industry, where product-development pressures create direct incentive to recruit needed expertise. Academic discovery research rarely has comparable resources and often loses experts to industry. Rebuilding this workforce is, therefore, a joint, complementary responsibility, with academic institutions providing the training environment and funders, such as the NIH, investing to sustain it as a matter of US research competitiveness. Expanding training opportunities in cross-species pathology and clinical medicine will increase the number of investigators capable of interpreting complex disease models rigorously. Further, integrating veterinary and human medical scientists, comparative pathologists and laboratory animal specialists more deeply into translational research teams will improve model selection, endpoint definition and phenotype interpretation.

This reintegration should also shape experimental design. Animal studies would benefit from clearer statements of model purpose, disease stage, species rationale, strain background and planned pathology-based phenotyping. Pathology is, too often, treated as a downstream confirmatory step rather than a primary analytic lens, even though phenotype is usually the first readout of experimental biology.

## Bridge the widening gap to advance science

Animal models remain necessary for discovery and system-level questions, especially when the relevant pathobiology is incompletely known. They allow investigators to move from whole-organism phenotype to organs, tissues, cells and molecules, and back again from mechanism to integrated physiology. This scientific function cannot yet be fully replaced by reduced systems alone. At the same time, the utility of animal models depends on how they are interpreted. The translational crisis in biomedical research reflects, at least in part, a crisis in comparative expertise in pathobiology and clinical medicine. If that hypothesis is correct, improving translation will require more than new technologies. It will require rebuilding the workforce of comparative experts needed to use *in vivo* systems to their full potential, while integrating NAMs where they are strongest. Artificial intelligence and computational tools can augment standardized phenotyping, but they cannot substitute for the context-dependent judgment of comparative experts when phenotypes are ambiguous or novel, particularly for behavioral abnormalities that can be easily misinterpreted. Bridging the gap between experimental models and human disease will, ultimately, depend on combining complementary NAMs with a deeper comparative understanding of animal models.
